# Using Sodium Thiosulfate to Heighten Copper (Cu (II)) Tolerance of the Freshwater Microalga *Chlorella vulgaris*

**DOI:** 10.3390/biology15030281

**Published:** 2026-02-04

**Authors:** Caihong Tian, Tongshun Si, Wenxin Chen, Menglin Liu, Zan Li, Weijun Wang, Guohua Sun, Yanwei Feng, Xiaohui Xu, Qiang Wang, Cuiju Cui, Jianmin Yang

**Affiliations:** 1 School of Fisheries, Ludong University, Yantai 264025, China; 2 School of Life Science and Bioengineering, Jining University, Jining 273155, China; 3 School of Engineering, Jining University, Jining 273155, China; 4 Shandong Engineering Research Center of Oyster Germplasm Creation and Efficient Culture, Yantai 264025, China

**Keywords:** *Chlorella vulgaris*, sodium thiosulfate, heavy metals, biochemical responses

## Abstract

Heavy metals such as copper are commonly found in aquatic environments. Microalgae can effectively adsorb heavy metals, while high concentrations impair their physiological and biochemical processes. This research investigated the impact of varying concentrations of sodium thiosulfate (Na_2_S_2_O_3_) on the heavy metal tolerance of *Chlorella vulgaris*. Results showed that Na_2_S_2_O_3_ and copper ions Cu(II) co-stress significantly improved the tolerance of *C. vulgaris* to Cu(II). To explore the mechanism, weighted gene co-expression network analysis (WGCNA) and trend analysis were applied to study the gene regulatory network under combined stress. A total of 103 significantly differentially expressed genes (DEGs) were identified. Further Gene Ontology (GO) and Kyoto Encyclopedia of Genes and Genomes (KEGG) analyses revealed that the majority of DEGs are associated with photosynthesis, energy and liposome metabolisms. Physiological metrics, including chlorophyll content, photosynthetic activity, malondialdehyde (MDA), superoxide dismutase (SOD), and catalase (CAT), also aligned with bioinformatics results. This research offers a promising approach to reduce heavy metal pollution in water bodies.

## Simple Summary

This study demonstrates that sodium thiosulfate (Na_2_S_2_O_3_) enhances copper (Cu(II)) tolerance in the microalga *Chlorella vulgaris*. Integrated gene expression and physiological analyses reveal that the improved tolerance involves coordinated regulation of key processes in photosynthesis, energy metabolism, and lipid metabolism. These findings provide a potential bioremediation strategy targeting copper-contaminated aquatic environments.

## 1. Introduction

Aquatic ecosystems, including wetlands, oceans, and lakes, are particularly prone to accumulating pollutants, especially heavy metals. Although heavy metal compounds primarily originate from natural sources, industrialization processes and agricultural activities, including metal mine drainage, metal-based pesticides, and aquaculture wastewater discharge, have often disrupted the balance of heavy metals in the environment [1]. Heavy metals such as Cu(II), Cr(VI), Zn, and Cd(II) are prominent water pollutants that pose risks to both aquatic ecosystems and human health even at trace levels [2,3]. Notably, many such metal compounds cannot be degraded by microorganisms and can accumulate in the food chain [4].

Cu(II) serves as a crucial transition element and an indispensable micronutrient that enhances the growth and metabolism of organisms at low concentrations; however, it can turn toxic at high concentrations [4]. Currently, several strategies, including physicochemical methods (such as membrane separation, ion exchange, chemical precipitation, and adsorption) and biological techniques (e.g., biosorption and bioprecipitation), are employed for Cu(II) remediation [2,5]. Furthermore, bacteria and microalgae have significant potential to remove Cu(II) from aquatic environments [6]. Compared to bacterial methods, microalgae excel in copper wastewater treatment by integrating remediation with resource recovery. They efficiently adsorb copper ions and the harvested biomass can be converted via hydrothermal liquefaction into biofuel, with copper stabilized in the solid residue to enable a circular economy [6]. Moreover, microalgae can generate valuable products such as lipids and pigments, and the harvested microalgal biomass can serve as a feedstock for biofuel production [6,7]. Thus, microalgae could be a cost-effective and ecologically safe solution for remediation of aquatic environments contaminated with Cu(II) [8]. Nonetheless, Cu(II) exposure has toxic effects on microalgae, including inhibiting growth, limiting photosynthesis, and impacting the composition of fatty acids [9]. Cu(II) can stimulate the production of reactive oxygen species (ROS), leading to oxidative damage to proteins, lipids, and thiol-peptides [1].

Tolerant species of microalgae are the preferred choices for Cu(II) bioremoval through bioaccumulation and biosorption [10]. Similarly, previous studies have demonstrated that dissolved organic matter, such as humic acids, plays a significant role in enhancing the bioaccumulation of metal ions by microalgae [11]. However, studies of inorganic salts remain scarce. In earlier investigations, the photosynthetic electron transfer rate and biomass yield of *Nannochloropsis oceanica* were effectively improved through pretreatment with sodium hydrosulfide (NaHS) that acts as a donor of H_2_S [12]. The findings indicated that low concentrations of H_2_S could significantly promote the synthesis of DNA and RNA, facilitate translation initiated by ribosomal proteins, and accelerate cell division [12]. Previous studies have demonstrated that Na_2_S_2_O_3_ not only improves *Chlorella* growth but also alleviates cadmium-induced oxidative stress to enhance plant tolerance [13,14]. Furthermore, it has been shown to stimulate lipid biosynthesis and increase biomass yield in microalgae [13]. Currently, there are no reports on Na_2_S_2_O_3_ enhancing the tolerance of *Chlorella* to heavy metals (e.g., Cu(II)).

In this study, *C. vulgaris* was utilized as a representative green microalga. The study aimed to evaluate the efficacy of Na_2_S_2_O_3_ in improving the tolerance of *C. vulgaris* against Cu(II) toxicity. To this end, different concentrations of Na_2_S_2_O_3_ were examined for their effects on the growth, chlorophyll content, free protein levels, and antioxidant enzyme pathways in green microalgae. Furthermore, a comparative analysis of the differentially expressed genes induced by the combined Na_2_S_2_O_3_ and Cu(II) stress was conducted using both WGCNA and trend analysis. The enhancement of heavy metal tolerance in *C. vulgaris* by Na_2_S_2_O_3_ has the potential to improve water bioremediation.

## 2. Materials and Methods

### 2.1. Microalgae Culture

*C. vulgaris* was sourced from the Freshwater Algae Culture Collection at the Institute of Hydrobiology (Code: FACHB-2338). The microalgae were cultivated following the method described previously [15]. They were cultivated in 1 L Erlenmeyer flasks containing 500 mL of BG11 basic medium at 25 ± 0.5 °C. The pre-culture was transferred into the culture medium, with an initial optical density at 680 nm (OD680) of 0.2. The culture medium was illuminated with cool white light-emitting diode (LED) lights (5800 ± 100 lux) under a 12 h light: 12 h dark photoperiod. The flasks were shaken three times daily to prevent the microalgae from adhering to the bottom of the flasks.

### 2.2. Biomass and Specific Growth Rate Measurement

The growth of microalgae was evaluated as described previously [15]. The dry weight of the microalgae has been found to correlate linearly with the absorbance of the culture at 680 nm using the following linear equation: y = 0.2898x + 0.01339 (R^2^ = 0.9919).

The specific growth rate μ, representing the microalgal cell proliferation rate and decay rate, was calculated using the following formula:

μ = (lnN_2_ − lnN_1_)/(T_2_ − T_1_)

where N_2_ and N_1_ represent the dry weights of microalgal biomass at times T_2_ and T_1_, respectively. T represents the period of growth (days).

### 2.3. Setting of Concentration Levels for Na_2_S_2_O_3_

Several concentrations of Na_2_S_2_O_3_ were selected (0, 0.05, 0.1, and 0.3 mmol/L) to assess the influence of Na_2_S_2_O_3_ on the tolerance of *C. vulgaris* to Cu(II). In this study, a blank group (0 mg/L Cu(II)), a control group (2 mg/L Cu(II)), and three experimental groups (2 mg/L Cu(II) with varying concentrations of Na_2_S_2_O_3_) were established. All experiments were carried out in triplicate. The incubation period lasted for 6 days, during which the relevant targets were measured daily.

### 2.4. De Novo Assembly and Gene Annotation

A total of twelve experimental samples, divided into four groups with three replicates each, were subjected to high-throughput sequencing on the Illumina HiSeq 4000 platform (LC-Bio Technology Co., Ltd., Hangzhou, China). Raw sequencing reads were processed with Cutadapt (v1.9) to eliminate undetermined bases, adapter contaminants, and low-quality bases (Q-score < 30). Subsequently, FastQC (v0.10.1) was employed for read quality assessment. The resulting high-quality clean data served as the foundation for all subsequent transcriptome analyses. De novo assembly was conducted using Trinity (v2.4.0) [16]. For functional annotation, the assembled transcripts were aligned against multiple databases using DIAMOND (v0.7.12) with an E-value cutoff of 1 × 10^−5^ [17]. The databases utilized included the Non-Redundant Protein Sequence Database (NR), Gene Ontology (GO), Kyoto Encyclopedia of Genes and Genomes (KEGG), Pfam, Swiss-Prot, and eggNOG.

### 2.5. Differential Expression Analysis

Gene expression levels were quantified using the Transcripts Per Million (TPM) method [18]. Differentially expressed genes (DEGs) were screened with the R package edgeR (v3.12.1), applying a threshold of absolute |log2 (fold change)| ≥ 1 and a *p*-value < 0.05.

### 2.6. Network Construction and Visualization

A weighted gene co-expression network was built with the WGCNA package (v1.70) in R using DEGs. A scale-free topology was achieved by applying a signed Spearman correlation model with a soft thresholding power (β) of 4. A hierarchical clustering dendrogram was constructed based on topological overlap matrix (TOM)-derived dissimilarity, and gene co-expression modules were identified using the dynamic tree cut algorithm, yielding 27 distinct modules.

### 2.7. Determination of Photosynthetic Activity

The in vivo chlorophyll fluorescence parameters were measured using multiple excitation wavelength phytoplankton and photosynthesis analyzer (PHYTO-PAM-II, Heinz Walz GmbH, Effeltrich, Germany). Two milliliters of microalgae was obtained and dark-acclimated for 20 min to prevent electron transfer between photosystems [19]. The efficiency of photosynthesis was quantified by assessing the maximum quantum yield, represented by the ratio Fv/Fm (Fv/Fm = (Fm − Fo)/Fm). Fo represents the minimum fluorescence of dark-adapted samples, Fm denotes the maximum fluorescence of dark-adapted samples, and Fv is the variable fluorescence, defined as Fm − Fo.

### 2.8. Determination of Photosynthetic Pigments

Photosynthetic pigments, including carotenoids and chlorophyll, were measured every day as a reference [19]. The microalgae were collected by centrifugation at 10,000 rpm for 10 min. The cell pellets were washed three times with 0.9% NaCl and resuspended in 2 mL of methanol (99.99%). The samples were then incubated in the dark at 4 °C for 24 h to determine the carotenoid and chlorophyll contents. The absorbances of the supernatant at 470, 652, and 665 nm were determined using a UV spectrophotometer (Infinite M200 Pro NanoQuant, TECAN, Grödig, Austria). The concentrations of chlorophyll a (Chl-a), chlorophyll b (Chl-b), and carotenoids (Caro) were calculated using the following formulas:

Chl-a (mg/L) = 16.72 × A_665_ − 9.16 × A_652_

Chl-b (mg/L) = 34.09 × A_652_ − 15.28 × A_665_

Caro (mg/L) = (1000 × A_470_ − 1.63 × Chl-a − 104.9 × Chl-b)/225

### 2.9. Determination of Soluble Protein and Antioxidant Activities

After 6 days of cultivation, the cell pellets were collected by centrifugation at 10,000 rpm for 10 min and then washed three times with 0.9% NaCl. The pellets were resuspended in 20 mmol/L phosphate-buffered solution (PBS) with a pH of 7.0 and lysed by sonication at 300 W for 15 min with an on/off ratio of 3 s/4 s. Malondialdehyde (MDA), catalase (CAT), and superoxide dismutase (SOD) were detected using the corresponding test kits (Nanjing Jiancheng Bioengineering Institute, Nanjing, China). The content of soluble protein was determined by the Coomassie Brilliant Blue G-250 method [20].

### 2.10. Statistical Analysis

An ANOVA was conducted to compare phenotypic traits. Prior to ANOVA, the assumptions of normality and homogeneity of variances were tested using the Shapiro–Wilk test and Levene’s test, respectively. The significance level was set at *p* ≤ 0.05. All statistical analyses were performed using SPSS 19.0 software (SPSS Inc., Chicago, IL, USA).

## 3. Results

### 3.1. Effects of Cu(II) and Na_2_S_2_O_3_ on the Growth of C. vulgaris

**Figure 1:**
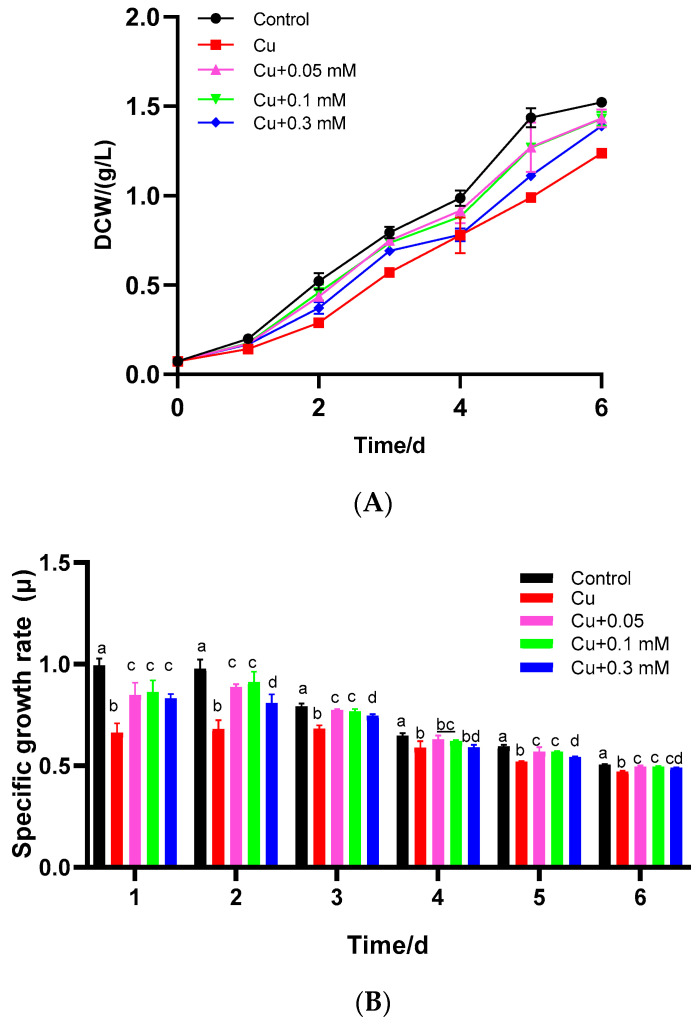
Growth characteristics of *C. vulgaris* under Cu(II) and different concentrations of Na_2_S_2_O_3_ in combination with 2 mg/L Cu(II): (**A**) *C. vulgaris* growth curve. (**B**) Average specific growth rate. The different lowercase letters in the columns imply that they are significantly different (*p* < 0.05).

The effects of Cu(II) alone and the combination of Cu(II) with different concentrations of Na_2_S_2_O_3_ on the density and specific growth rate of *C. vulgaris* were evaluated. Compared to the blank group (*p* < 0.05), the specific growth rate of *C. vulgaris* was significantly suppressed when the algae were exposed to 2 mg/L CuSO_4_, exhibiting a 33.3% reduction on the first day (Figure 1A,B). After 6 days of cultivation, the biomass of the group treated with 2 mg/L CuSO_4_ was decreased by approximately 20%. These results indicated that Cu(II) had a toxic effect on *C. vulgaris*, inhibiting microalgal cell growth. Subsequently, different concentrations of Na_2_S_2_O_3_ were added to verify their effect on improving the tolerance of *C. vulgaris* to Cu(II). Compared to the control group, each experimental group with a different dosage of Na_2_S_2_O_3_ showed varying degrees of growth promotion on *C. vulgaris*. The increase in the logarithmic growth rate was negatively correlated with the increasing concentration of Na_2_S_2_O_3_. Compared to the Cu(II) control, growth rates were significantly elevated with 0.05 and 0.1 mM Na_2_S_2_O_3_ (*p* < 0.05), although the difference between these two concentrations was not significant (*p* > 0.05). However, at 0.3 mM Na_2_S_2_O_3_, the growth rate decreased significantly (*p* < 0.05).

### 3.2. RNA-Seq Analysis, Gene Co-Expression Network, Trend Analysis, and Functional Enrichment of Overlapping Genes

The generated raw reads were assembled using Trinity. 55,407 unigenes and 148,065 transcripts were generated, with a total length of 59,442,498 bases and an N50 of 2131 (Table A1). All predicted genes were functionally annotated against multiple databases using DIAMOND; the detailed annotation results are summarized in Table A2.

**Figure 2:**
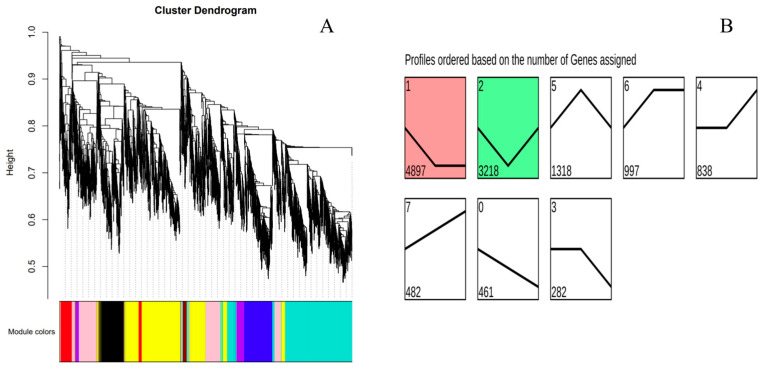
Clustering dendrogram (**A**) and trend analysis (**B**) of differentially expressed genes.

WGCNA identified a total of 27 functional modules from the gene co-expression network constructed with a soft threshold of 9 (Figure 2A). Among these, five modules—royalblue, black, tan, yellow, and red—showed strong correlations with Na_2_S_2_O_3_ concentration. Trend analysis performed on the 12,493 DEGs identified eight distinct expression trends, among which Trend 1 (4897 genes, initially downregulated then stabilized) and Trend 2 (3218 genes, initially downregulated then upregulated) showed significant correlations with Na_2_S_2_O_3_ concentration (*p* < 0.05) (Figure 2B).

**Figure 3:**
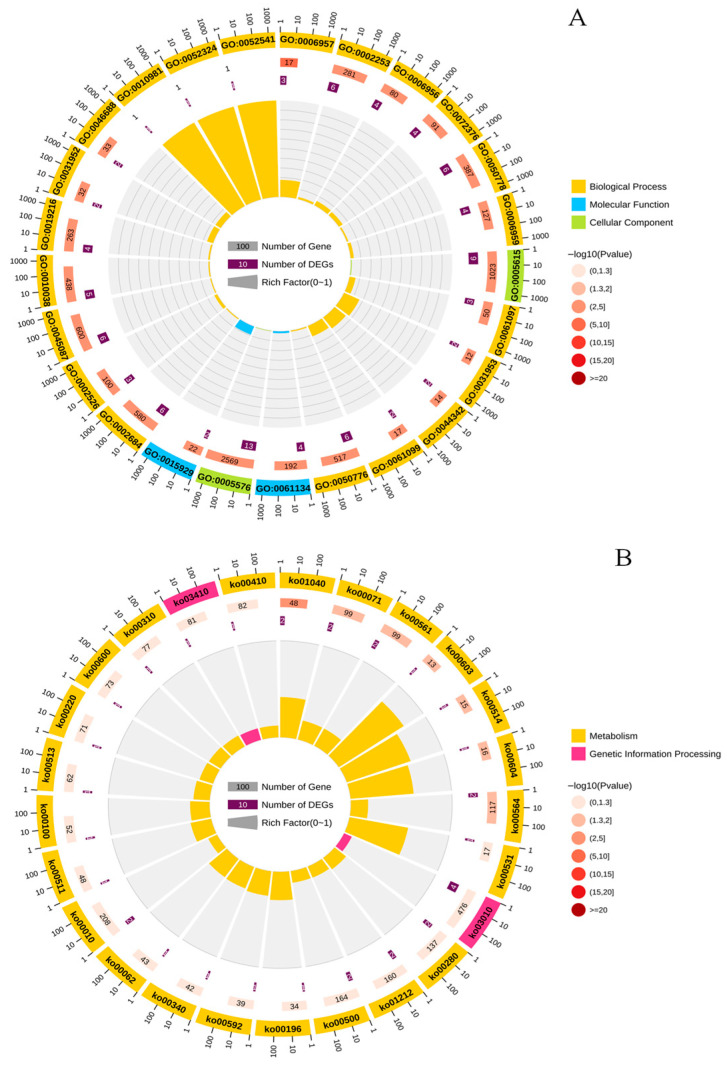
GO (**A**) and KEGG (**B**) enrichment analysis of intersecting target genes. The size of the circle represents the number of genes, and the color of circle represents the enrichment level indicated by the *p* value.

**Figure A1:**
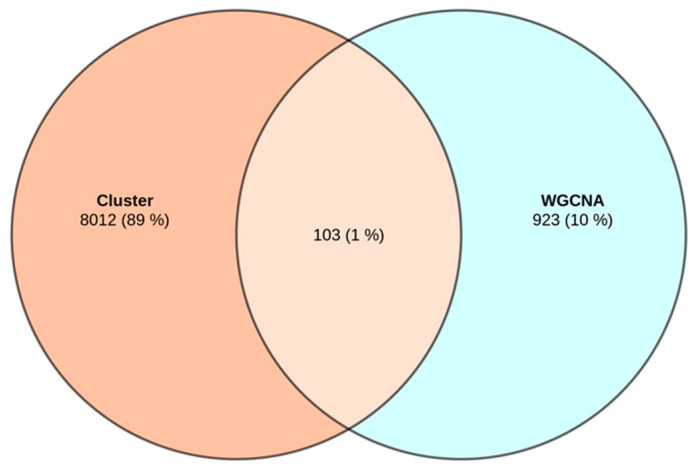
Venn diagram of common and unique differentially expressed genes (DEGs) displayed by trend analysis and WGCNA analysis. Orange represents the differentially expressed genes from trend analysis; light blue represents the differentially expressed genes from WGCNA.

A Venn diagram revealed 103 overlapping genes common to both WGCNA and trend analysis (Figure A1). GO enrichment analysis of these 103 genes revealed that the majority of significant terms were associated with immune regulation, metal ion stress response, and protein autophosphorylation (Figure 3A). KEGG enrichment analysis indicated that the combined stress of Cu(II) and Na_2_S_2_O_3_ affected multiple metabolic pathways in *C. vulgaris*, including photosynthesis-antenna proteins, fatty acid metabolism, amino acid metabolism, and glycolysis/gluconeogenesis (Figure 3B). Finally, a total of 14 key differentially expressed genes (DEGs)—including Elovl5, Aldh7a1, hexa1, HPAT1, Scp2, GPAT, GDPD6, Cfh, Prnp, CAV1, C3, Errfi1, Cfb, and C4b— were ultimately identified as involved in the toxicological response.

### 3.3. Effects of Cu(II) and Na_2_S_2_O_3_ on Chlorophyll Content

**Figure 4:**
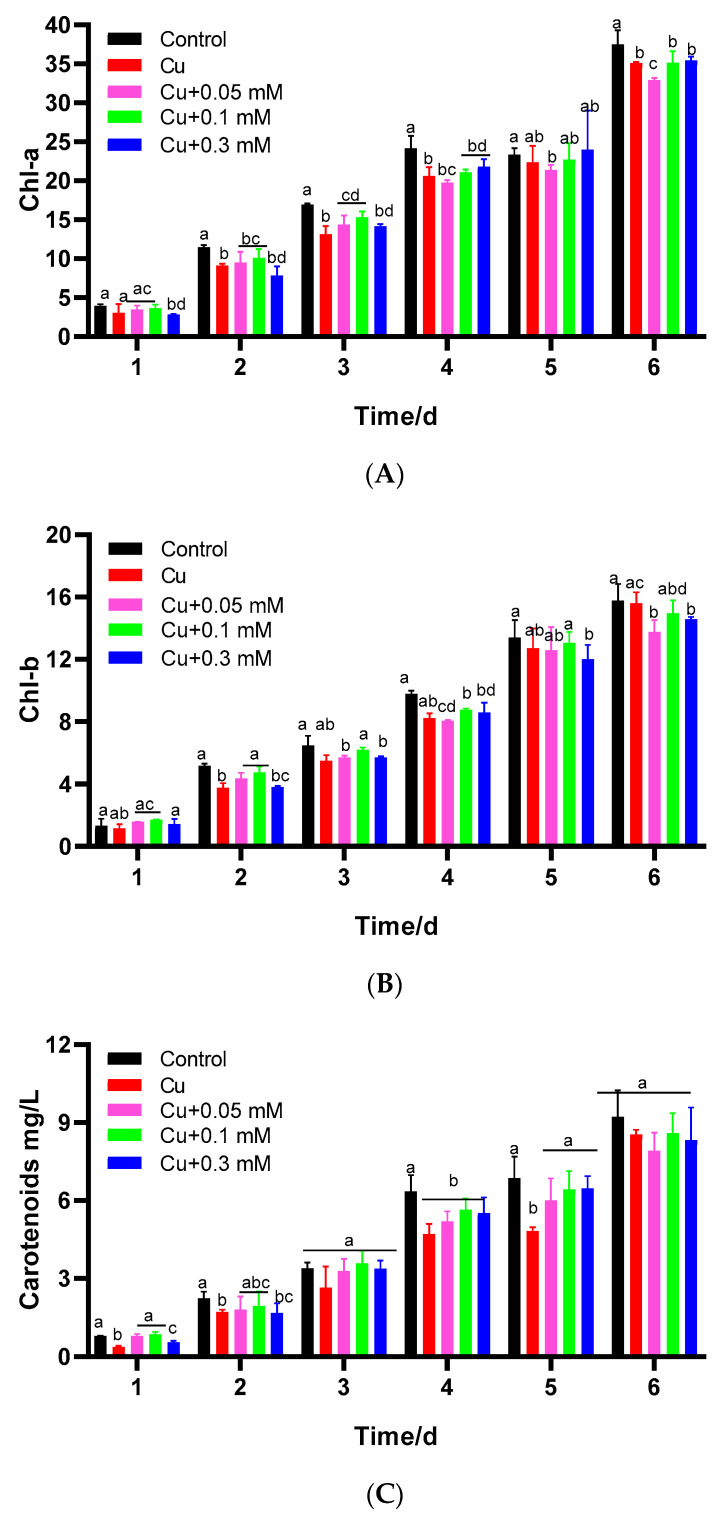
Effects of Cu(II) and different concentrations of Cu(II) + Na_2_S_2_O_3_ on the contents of chlorophyll a (**A**), chlorophyll b (**B**), and carotenoids (**C**) in *C. vulgaris* (mg/g) after 6 days of cultivation. The different lowercase letters in the columns imply that they are significantly different (*p* < 0.05).

The chlorophyll a content was significantly decreased by the addition of 2 mg/L Cu(II) throughout the cultivation period compared to the blank group (*p* < 0.05) (Figure 4). In contrast, the differences observed in chlorophyll b and carotenoid contents were less pronounced. When Na_2_S_2_O_3_ was added, the concentrations of chlorophyll a and chlorophyll b were restored compared to the Cu(II)-only control group. A negative correlation was observed between the effects of 0.1 mM and 0.3 mM Na_2_S_2_O_3_ in the presence of Cu(II) within the first 3 days. With the extension of culture time, the concentrations of chlorophyll a and carotenoids rose progressively, corresponding to the increasing concentration of Na_2_S_2_O_3_, eventually reaching a stable state. On the sixth day, the difference in chlorophyll a was 5.4%, and the carotenoid content was increased by 4.1%.

### 3.4. Effects of Cu(II) and Na_2_S_2_O_3_ on Fv/Fm

**Figure 5:**
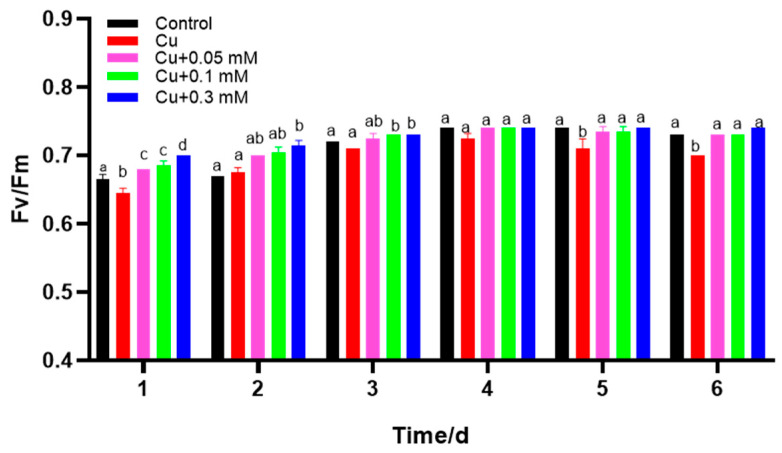
Cu(II) and Cu(II) + Na_2_S_2_O_3_ induced variation in the maximum quantum efficiency (Fv/Fm). The different lowercase letters in the columns imply that they are significantly different (*p* < 0.05).

The addition of Cu(II), particularly in the early stages, significantly decreased the Fv/Fm values of *C. vulgaris* (Figure 5). In contrast, the Fv/Fm values were significantly increased in the Na_2_S_2_O_3_ treatment groups during the initial two days of culture. Notably, higher concentrations of Na_2_S_2_O_3_ were associated with a more pronounced enhancement of Fv/Fm. In the later stages of culture, both the control and Na_2_S_2_O_3_-treated groups maintained elevated Fv/Fm levels.

### 3.5. Effects of Cu(II) and Na_2_S_2_O_3_ on Soluble Protein and Antioxidant Enzyme Activity

**Figure 6:**
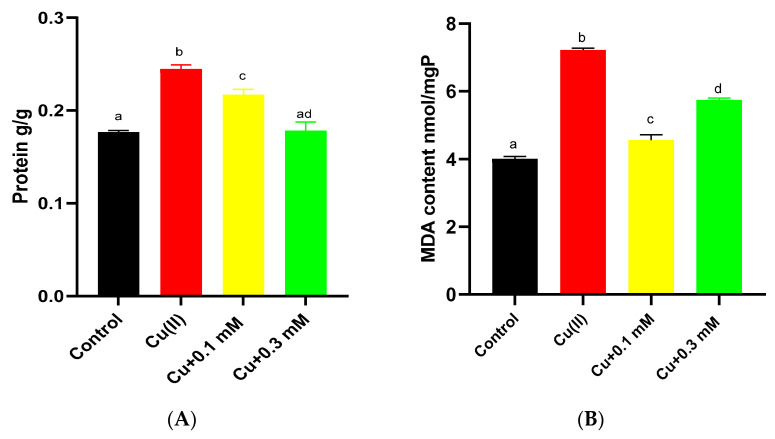

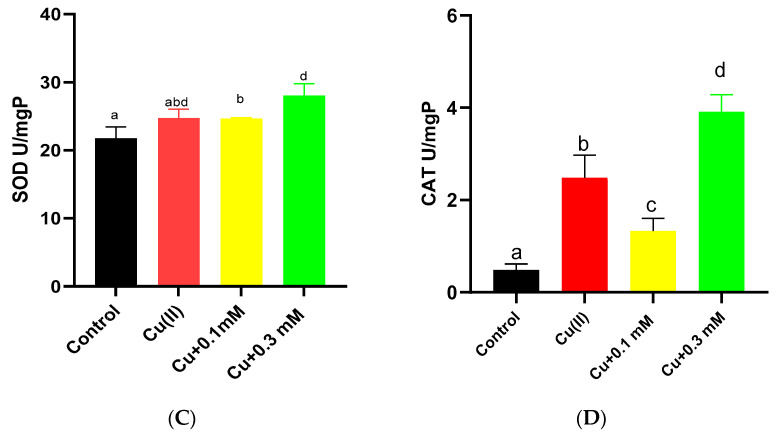
Concentrations of (**A**) soluble protein, (**B**) MDA, (**C**) SOD, and (**D**) CAT of *C. vulgaris* under treatment with Cu(II) and different concentrations of Na_2_S_2_O_3_ in combination with 2 mg/L Cu(II). The different lowercase letters in the columns imply that they are significantly different (*p* < 0.05).

When subjected to Cu(II) stress for 6 days, the soluble protein content increased from 0.17 g/g dry weight to 0.24 g/g dry weight (Figure 6A). In experiments involving Na_2_S_2_O_3_, the protein contents gradually decreased. At dosages of 0.1 mM and 0.3 mM Na_2_S_2_O_3_, the protein contents were decreased by 11.2% and 27.2%, respectively.

The content of MDA was significantly increased upon exposure to Cu(II) (*p* < 0.05) (Figure 6B). When Na_2_S_2_O_3_ was added in conjunction with Cu(II), the MDA content decreased. Notably, the application of 0.3 mM Na_2_S_2_O_3_ resulted in higher MDA levels compared to the 0.1 mM treatment.

The enzymatic activities of SOD and CAT increased by 13.7% and 408%, respectively, when exposed to Cu(II) (Figure 6C,D). Under low concentrations of Na_2_S_2_O_3_ (0.1 mM), the activities of SOD and CAT were 24.63 U/mg and 0.28 U/mg, respectively, showing varying degrees of reduction compared to the Cu(II)-only control values of 24.71 U/mg and 0.50 U/mg. However, when the concentration of Na_2_S_2_O_3_ was increased to 0.3 mM, both SOD and CAT activities increased to 28.03 U/mg and 3.91 U/mg, respectively.

## 4. Discussion

The results demonstrate that Cu(II) exerted significant toxic effects on *C. vulgaris*, inhibiting growth, reducing chlorophyll a content, and impairing PSII photochemical efficiency (Fv/Fm). This toxicity is likely attributable to Cu(II) catalyzing ROS generation via Fenton and Haber-Weiss reactions, leading to cellular damage [21,22]. The observed reduction in chlorophyll a, a common stress response in microalgae, may be linked to ROS-induced thylakoid lipid peroxidation and degradation of the PSII complex [23,24,25,26].

The addition of Na_2_S_2_O_3_ exhibited a concentration-dependent dual effect. At low concentrations (0.05 and 0.1 mM), Na_2_S_2_O_3_ significantly promoted the growth of *C. vulgaris* under Cu(II) stress, restored photosynthetic pigment content, enhanced Fv/Fm, and reduced oxidative damage markers (MDA) and the activities of antioxidant enzymes (SOD, CAT). This indicates that low-concentration Na_2_S_2_O_3_ alleviates Cu(II) toxicity potentially by enhancing photosynthetic pigment synthesis, maintaining efficient photosynthetic electron transfer [12], and mitigating oxidative stress. Carotenoids likely contributed to this protection by regulating chlorophyll and quenching reactive oxygen species [27,28,29].

However, at a higher concentration (0.3 mM), Na_2_S_2_O_3_ inhibited the specific growth rate, led to higher MDA levels, and induced increased activities of SOD and CAT. This suggests a potential intrinsic oxidative damage caused by Na_2_S_2_O_3_ itself at elevated levels, which counteracts its protective role and ultimately inhibits algal growth [12,30].

Transcriptomic analysis provided deeper mechanistic insights. The 103 overlapping genes identified by both WGCNA and trend analysis are key regulators responding to Na_2_S_2_O_3_ concentration. Their enrichment in GO terms related to immune regulation, metal ion stress, and protein autophosphorylation, and in KEGG pathways such as photosynthesis-antenna proteins, fatty acid metabolism, and central carbon metabolism (glycolysis/gluconeogenesis, pyruvate metabolism, and the TCA cycle), indicates that the combined stress perturbs these core metabolic processes. The susceptibility of the light-harvesting complex function to stress, the disruption of lipid synthesis, and the pivotal role of carbon metabolism in stress response offer molecular explanations for the observed physiological changes in photosynthesis and growth [31,32,33]. At the same time, five core genes involved in cell wall synthesis, aldehyde detoxification, and lipid metabolism (GDPD6, HPAT1, ALDH7A1, GPAT, and Scp2) changes significantly, collectively regulating the stress response in microalgae. Among them, GDPD6 helps maintain cell wall strength by influencing cellulose synthesis [34]; ALDH7A1 alleviates oxidative damage by catalyzing the dehydrogenation of toxic aldehydes [35]; HPAT1 participates in cell wall protein modification to enhance structural integrity [36]; GPAT regulates lipid metabolism and membrane fluidity [37]; and Scp2 is involved in lipid transport and signal transduction [38]. Working synergistically across multiple aspects—including cellular structural stability, toxin clearance, and membrane function maintenance—these genes jointly improve the tolerance and adaptability of microalgae under stress conditions.

The increase in soluble protein under Cu(II) stress may be part of a detoxification mechanism (e.g., metal complexation) [9,39,40] or a general stress response [19,39]. Its subsequent decrease with Na_2_S_2_O_3_ addition suggests a mitigation of oxidative stress. The pattern of antioxidant enzyme activities reinforces the oxidative stress dynamics: induction by Cu(II) signifies high ROS levels; reduction by low-concentration Na_2_S_2_O_3_ indicates alleviated stress; and re-induction at high Na_2_S_2_O_3_ concentration points towards renewed oxidative pressure.

## 5. Conclusions

In this study, we investigated the effect of Na_2_S_2_O_3_ on enhancing the tolerance of *C. vulgaris* to Cu(II). Cu(II) can inhibit the growth of *C. vulgaris*, reduce its photosynthetic efficiency, and cause photodamage. The addition of Na_2_S_2_O_3_ in combination with Cu(II) had positive impacts on the growth and proliferation of *C. vulgaris*. At low concentrations, Na_2_S_2_O_3_ enhanced the tolerance of *C. vulgaris* to Cu(II), promoted chlorophyll content conversion, and aided in mitigating the toxicity associated with Cu(II) pollution. Furthermore, Na_2_S_2_O_3_ improved the Fv/Fm ratio under Cu(II) stress while reducing MDA levels as well as the activities of SOD and CAT. The Cu(II)- Na_2_S_2_O_3_ complexes make *C. vulgaris* an excellent organism for the potential bioremediation of this heavy metal. Transcriptomic analysis further revealed that the combined stress of Cu(II) and Na_2_S_2_O_3_ perturbs key metabolic pathways in *C. vulgaris*, including those related to photosynthesis, fatty acid metabolism, and carbon metabolism, highlighting the complex molecular adjustments underlying the algal response. These results suggest that Na_2_S_2_O_3_ can enhance microalgal resistance to Cu(II). However, the mechanisms underlying this enhanced metal resistance, particularly at low concentrations of Na_2_S_2_O_3_, remain unclear and warrant further investigation.

## Data Availability

The raw data supporting the conclusions of this article will be made available by the authors on request.

## Abbreviations

The following abbreviations are used in this manuscript

ANOVA	Analysis of Variance
DEGs	Differentially Expressed Genes
GO	Gene Ontology
KEGG	Kyoto Encyclopedia of Genes and Genomes
LMW	Low Molecular Weight
PBS	Phosphate-Buffered Solution
ROS	Reactive Oxygen Species
TPM	Transcripts Per Million
WGCNA	Weighted Gene Co-expression Network Analysis
CAT	Catalase
Cd	Cadmium
Cu(II)	Copper ions
H_2_S	Hydrogen Sulfide
MDA	Malondialdehyde
Na_2_S_2_O_3_	Sodium Thiosulfate
SOD	Superoxide Dismutase
Caro	Carotenoids
Chl-a	Chlorophyll a
Chl-b	Chlorophyll b
Fv/Fm	Maximum Quantum Yield of Photosystem II
OD680	Optical Density at 680 nm
PSII	Photosystem II
TCA cycle	Tricarboxylic Acid Cycle

## Appendix A

**Table A1:** The Trinity assembly results.

Index	All	GC%	Min Length	Median Length	Max Length	Total Assembled Bases	N50
Transcript	148,065	56.43	180	1630	35,853	334,014,254	3721
Gene	55,407	55.68	201	506	35,853	59,442,498	2131

**Table A2:** Diamond annotation results.

Database Annotation Results	Numbers	Ratio (%)
All	55,407	100.00
GO	21,504	38.81
KEGG	13,032	23.52
Pfam	19,697	35.55
swissprot	20,343	36.72
eggNOG	23,879	43.10
NR	18,408	33.22
TF	240	0.43
